# Period Prevalence and Sociodemographic Factors of Hypertension in Rural Maharashtra: A Cross-Sectional Study

**DOI:** 10.4103/0970-0218.55269

**Published:** 2009-07

**Authors:** Sampatti Sambhaji Todkar, Venktesh V Gujarathi, Vinay S Tapare

**Affiliations:** Department of PSM, Dr. V.M.Govt. Medical College Solapur, 413 003, India; 1 Department of PSM, GMC, Aurangabad, India

**Keywords:** Hypertension, prevalence, BMI

## Abstract

**Background:**

Hypertension is most common cardiovascular disease and it account for large proportion of all cardiovascular deaths and disability worldwide.

**Research Questions:**

What is the level of prevalence of hypertension in rural area? What are the soociodemographic factors associated with hypertension?

**Objectives:**

To find out prevalence of hypertension in rural area.

**Study Design:**

A community-based cross-sectional study setting: Rural Health Training Centre Paithan, field practice area of govt. medical college Aurangabad, Maharashtra.

**Participants:**

1297 persons aged 19 years and above.

**Study Period:**

June 2005 to December 2006.

**Materials and Methods:**

A house-to-house survey was conducted by the author himself, interviewed the participants by systematic random sampling method, using pretested structured standard questionnaire. Two independent blood pressure (BP) readings were taken in sitting position by visiting each participant at their home. Hypertension was defined as systolic BP more than or equal to 140 mm of Hg or diastolic BP more than or equal to 90 mm of Hg or those individuals currently taking antihypertensive treatment.

**Statistical Tests:**

Percentiles, Chi Square test, Chi-Square for linear trend, multiple logistic regression analysis on SPSS software Version 10.

**Results:**

Overall prevalence of hypertension in the study subjects was 7.24%. Multiple logistic regression analysis identified various factors significantly associated with hypertension were age, sex, BMI, additional salt intake, smoking, DM, alcohol consumption, and higher socioeconomic status.

**Conclusions:**

The overall prevalence of hypertension in study subjects was 7.24%.

## Introduction

Hypertension is the most common cardiovascular disease, emerging as a major public health problem in developing as well as developed countries. The WHO report 1998 states that considering the prevalence of any disease, hypertension ranks forth in the world.(1) Pooled epidemiological studies show the average prevalence of hypertension in India is 25% in Urban and 10% in rural population. Hypertension is a significant public health problem in urban and rural areas of India. It is directly responsible for 57% of all stroke deaths and 42% of coronary heart disease death in India. It is also a leading cause of blindness, renal failure and congestive heart failure.(1,2)

Because of the changing life styles, the environment, industrialization, and urbanization the prevalence of hypertension is increasing constantly. Field-based studies on the prevalence of hypertension are still scarce and more fields based are required to highlight problem of hypertension. Hence this field based cross-sectional study was undertaken.

## Materials and Methods

Narala ward of Paithan was selected by lottery method. The sampling fraction was 30.51% (1297 of 4250). The required sample size was 1233 based on the prevalence of hypertension 7.5% as observed in the pilot study. Narala was having 1024 houses with total population of 4250 (19 years and above). A house-to-house survey was conducted by systematic random sampling method and a total 1297 persons of 19 years and above from 256 houses (every forth household was selected in the study sample) were interviewed, and detailed information regarding age, sex, educational status, occupation, type of family, literacy status, marital status and personal habits like smoking, alcohol intake and additional dietary salt consumption was collected. Of the 1360 usual residents in the study area 63 (4.85%) were excluded due to reasons like non-availability in spite of three successive visits, unusual residents, and refusal to get examined. The overall response rate was 95.15%.

The hypertension was defined according to Fifth report Joint National Committee for detection, evaluation and treatment of high blood pressure, as systolic BP more than or equal to 140 mm of Hg or diastolic blood pressure more than or equal to 90 mm of Hg or those individuals currently taking antihypertensive treatment.(2) Blood pressure was measured by mercury sphygmomanometer, pulse obliteration and auscultation method in sitting position. The average interval between two BP readings was at least 10 minutes. The BP measurements were done strictly as per WHO criteria's, the mean of two readings was used for analysis.

## Results

The age wise distribution of study subjects along with prevalence of each group is shown in Table 1, out of 1297 study subjects examined, 94 (7.24%) were having hypertension.

**Table 1:** Age-wise prevalence of hypertension among study subjects

Age group	Hypertensives N (%)	Normotensives N (%)	Odds ratio (OR)	95% C.I. of O.R.
19-28	02 (0.41)	482 (99.59)	01.00	-
29-38	07 (2.56)	266 (97.44)	06.34	01.20 - 44.46
39-48	14 (6.86)	190 (93.14)	17.76	03.81 - 114-19
49-58	26 (16.77)	129 (83.23)	48.57	11.00 - 300.05
59-68	26 (23.63)	84 (76.37)	74.60	16.75 - 463.57
69-78	14 (25.45)	41 (74.05)	82.29	16.96 - 544.10
≥ 79	05 (31.25)	11 (68.75)	109.55	16.09 - 934.70
Total	94 (7.24)	1203 (92.76)	-	-

χ^2^ = 148.30, DF = 6, P < 0.001; highly significant, figures in parenthesis are in percentages

**Figure 1:**
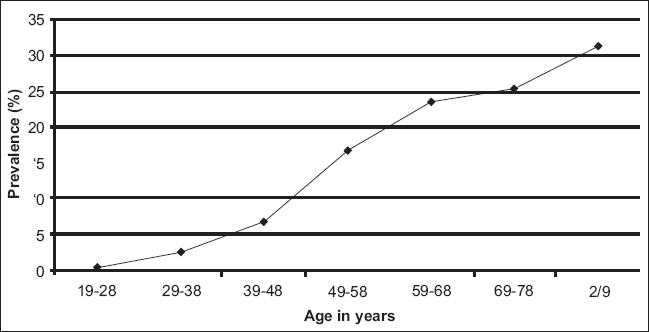
Line diagram showing the relationship between prevalence of hypertension and age

The prevalence of hypertension was increased significantly with increasing age. The lowest prevalence of hypertension was 0.41% in the age group of 19-28 years, and highest prevalence of hypertension was 31% in the age group of more than or around 79 years [Figure 1]. Highly statistically significant association (*P* < 0.001) was observed between age group and hypertension.

Table 2 shows the univariate analysis of important risk factors associated with hypertension.

**Table 2:** Study variables and its association with hypertension: Univariate analysis

Variables		Hypertensives N (%)	Normotensives N (%)	Odds ratio	95% C.I. of O.R
Sex	Males	42 (44.68)	599 (49.79)	0.81*	0.79 - 1.91
	Females	52 (55.22)	604 (50.21)	-	
BMI	≤ 18.5	10 (0.034)	280 (99.66)	1	-
	18.6-24.9	52 (5.58)	861 (94.42)	1.69*	0.82 - 3.59
	25.0 - 29.9	26 (34.21)	53 (65.79)	13.74	5.91 - 32.59
	≥ 30	06 (40.00)	09 (60.00)	18.67	4.77 - 73.59
Salt intake	Yes	20 (28.99)	49 (71.01)	6.36	3.46 - 11.66
	No	74 (6.02)	1154 (93.98)		
Alcohol intake	Yes	08 (28.57)	20 (71.42)	5.5	2.16 - 13.65
	No	86 (6.77)	1183 (93.22)		-
	Yes	14 (60.86)	09 (13.13)	23.21	9.10 - 60.21
	No	80 (6.75)	1194 (93.24)		-

* Not significant, figures in parenthesis are in percentages

**Figure 2:**
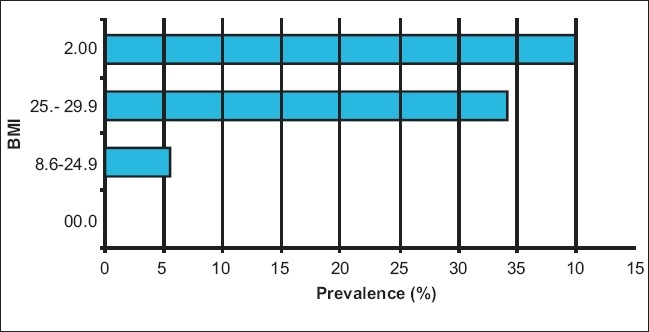
Bar diagram showing prevalence of hypertension according to body mass index

Though the prevalence of BP in males (44.68%) was less compared to females (55.22%), it was not statistically significant (*P* > 0.05). About 70.39% study participants were in the range of normal BMI (18.6-24.9), of these 5.58% were having hypertension though the risk of hypertension is more in this group as compared to group having BMI < 18.5 it was statistically not significant. But the subjects having BMI more than or equal to 25 were definitely having significantly higher prevalence of hypertension as compared to group of BMI less than 18.5 (OR= 1.69 95% of CI 0.82- 3.59) [Figure 2]. The distribution of study subjects according to their dietary salt intake reveals the overall response rate for the additional dietary salt intake was 5.31%. Of these 28.99% were having hypertension.

The additional dietary salt intake was defined as those individuals who ate more then two pinches of salt per meal excluding the previously added salt to meal during preparation,(3) the prevalence of hypertension was significantly higher in additional dietary salt consumers as compared to those not taking extra salt. (OR = 6.36, 95% CI 3.46 - 11.66) Association between alcohol intake and hypertension shows 2.15% were having the habit of alcohol consumption of these 28.57% study subjects found with hypertension. 5.5 times higher risk was observed in alcohol consumers (OR = 5.5). 1.77% study subjects were having Diabetes Mellitus; of these 60.86% were having hypertension and 39.14% not having hypertension (OR 23.21).

Multiple logistic regression analysis revealed the following factors, was significantly associated with hypertension, and as far as the individual role of each factor concerned, after controlling for other variables revealed that the ‘age’ 21.97% for SBP and 12.95% for DBP) as the most important risk factor for hypertension [Table 3]. Then the second most important factor came out to be BMI (3.85% for SBP and 4.5% for DBP), other factors significantly associated was BMI, education, DM, smoking, socioeconomic status, alcohol intake, history of hypertension in parents and history of hypertension in family.

**Table 3:** Independent contribution (after controlling other variables) by each regressor variable to the SBP and DBP (hypertension) (n = 1297)

Variable	SBP			DBP		
		
	R^2^ change%	F change	*P* value	R^2^ change%	F change	*P* value
Age	21.97	285.77	**	12.95	168.74	**
BMI	3.85	50.55	**	4.50	59.03	**
Salt intake	3.31	43.09	**	2.85	37.11	**
Education	1.77	23.90	**	1.38	18.16	**
DM	1.76	23.43	**	1.38	18.23	**
Smoking	1.38	18.32	**	0.84	11.28	**
S.E. status	0.9	12.23	**	0.15	02.00	*
Alcohol	0.46	06.32	*	0.69	09.69	*
Occupation	0.15	02.69	*	0.06	0.85	NS
h/o H. parents	0.07	01.36	*	0.38	05.88	*
f/h of Htn	0.01	0.14	NS	0.23	03.69	*

NS: Not Significant

* P < 0.05

** P < 0.001, H/o H. parents = history of hypertension in parents, F/h of htn. = family history of hypertension

## Discussion

The overall prevalence of hypertension in the study subject was 7.24% (94 of 1297). The prevalence of hypertension was increased gradually with the increasing age i.e. it was maximum (31.25%) in age group of 79-90 years [Table 1], while it was minimum i.e. (0.41%) in age group of 19-28 years. The odds ratio was found significantly increasing gradually with increasing age i.e. from one (19-28years) to 109 (more than 79 years).

The similar findings have been reported by various studies in India and in other countries too e.g. Joshi S V *et al.* 2000, from Bombay hospital, Mumbai reported 7.82% prevalence of hypertension (6.1% and 10.5% in males and females respectively).(4) Jajoo UN (1993) *et al*. from Sevagram reported 3.41% prevalence in rural population.(5)

Of the total 94 subjects with hypertension, 42 (6.55%) were males and 52 (7.92%) were females having hypertension, i.e. a higher hypertension prevalence rate was observed in females as compared with males. However, this difference was statistically not significant. Comparable findings reported by Jajoo (1993) *et al*. from Sevagram was 2.9% and 4.6% in males in females respectively.(5)

In comparison with studies done outside India, the overall prevalence rate observed in the present study is low, e.g Pitsavos *et al.*,(6) and Sans *et al*.,(7) observed 15.5% and 15.0% prevalence respectively. However, the arterial BP levels in Asian people are lower as compared with western world and the possible differences may be due to genetic and environmental factors like, stress, fast food, climate etc The rise of blood pressure with age is said to be due to aging process, atherosclerotic changes in blood vessels, stress and strain and unknown factors.

A positive association was observed between body mass index and development of hypertension. Persons having BMI more than or equal to 25 were definitely having higher risk of hypertension. The similar findings were reported by number of epidemiological studies e.g. Jajoo(6) 1993, Das *et al.* 2005,(8) Malhotra *et al.* 1998.(9)

Since a long time, extra salt intake has been considered to cause hypertension. This was proved true in this study and in other studies too. The hypertension prevalence was higher (28.99%) in participants following additional dietary salt intake compared to those who gave negative history of additional dietary salt consumption. A 6.36 times higher risk was found in study subjects with additional salt intake, as compared with non-additional salt consumers. Similar association between salt and hypertension has been observed by Singh,(10) and Sadhukhan.(11)

A direct correaltion was found between alcohol intake and hypertension. Higher prevalence of hypertension was observed among study subjects with history of alcohol consumption as compared with the study subjects with no history of alcohol consumption. 5.5 times higher risk for development of hypertension was observed in alcohol consumers (OR = 5.5). Alcohol as a positive risk for development hypertension has also observed by Singh,(10) and Sadhukhan.(11)

It is said that hypertension and diabetes go hand in hand and this was found true, 1.77% study subjects was found with Diabetes Mellitus; of these 60.86% were having hypertension and 39.14% not having hypertension. Higher prevalence of hypertension was observed among study subjects with Diabetes Mellitus as compared with non-diabetics. 23.21 times higher risk was found in diabetics. (Table 2, OR = 23.21). This association has been supported by various studies from India and other countries also.(12)

The multiple linear regression analysis was performed for SBP and DBP with the assumption that the factors responsible for hypertension will also be responsible for SBP and DBP, as there is no natural dividing line between normal and high blood pressure. To perform multiple logistic regression analysis, all the variables were assigned uniform numerical scores.(13) Multiple logistic regression analysis reveled that the age (21.97%) as a single most important risk factors for hypertension. Other factors significantly associated with hypertension were BMI, extra salt intake, diabetes mellitus, smoking, alcohol consumption, history of hypertension in parents and family of history of hypertension in decreasing order.

Similar findings was observed by Sadhukhan *et al*. and Bagchi(14) *et al*., Sadhukhan in a prevalence study in adults aged 18 years and above in a Singur block of Hoogly district of west Bengal found the age as most important factor (16.57% for SBP and 7.90% DBP), BMI (3.10% for SBP and 7.78% for DBP), occupation (3.18% for SBP and 0.66% for DBP), and additional salt intake (3.31% for SBP and 2.85% for DBP).

## Conclusion

The overall prevalence of hypertension in the study subjects was 7.24%. The prevalence of hypertension increased gradually with increase in age, BMI, additional salt intake, alcohol consumption and with diabetes mellitus.

## Recommendations

The increasing trend of prevalence of hypertension with increasing age strongly suggest us to prepare and implement a community based ‘high risk’ screening program to prevent the modern epidemic of chronic non-communicable diseases like hypertension.

‘High risk’ screening programme should be started to detect persons at risk of developing hypertension.

Information, education, and communication activities (IEC) should be started to increase the awareness of people to adopt healthy life style, slike regular physical exercise, restricted salt intake, avoidance of alcohol and smoking.
